# 4131 Recruitment and Retention of Individuals with a Cocaine Use Disorder

**DOI:** 10.1017/cts.2020.279

**Published:** 2020-07-29

**Authors:** Kate Brown, Bernadette Capili

**Affiliations:** 1 Rockefeller University

## Abstract

OBJECTIVES/GOALS: 1) illustrate the varied challenges individuals with a cocaine use disorder experience in daily life, 2) demonstrate techniques for empathizing and building rapport with potential subjects, and 3) identify recruitment obstacles and solutions. METHODS/STUDY POPULATION: Methods: We use a multi-source strategy to recruit our participants and employ practical techniques to enhance protocol adherence. Methods include a welcoming environment, establishing a routine with flexibility, personalized attention, and incentives for participation. Study population: Individuals with a cocaine use disorder. RESULTS/ANTICIPATED RESULTS: Understanding the life of an individual with a cocaine use disorder is paramount to successful recruitment and retention in addiction research studies. Our clinicians have been able to recruit and retain participants successfully by employing empathetic interpersonal skills, personalized attention, and health-related incentives. DISCUSSION/SIGNIFICANCE OF IMPACT: The Centers for Disease Control and Prevention estimated that 69,029 people died of a drug overdose during the period from February 2018 to February 2019, with 23%, due to cocaine. While methadone and buprenorphine-naloxone maintenance treatment allow opioid-dependent individuals achieve a sense of physical and mental stability, there is no pharmaceutical treatment to help a cocaine-dependent individual cope with cravings or the depression and anxiety that typically follow a cocaine binge. The development of a cocaine use disorder is multi-factorial and presents a significant challenge in terms of discovering treatments, identifying efficient recruitment and retention strategies is the first step for effective research.

